# Prevalence and correlates of diabetes and metabolic syndrome in a rural indigenous community in Baja California, Mexico

**DOI:** 10.1186/s12889-018-6276-x

**Published:** 2018-12-20

**Authors:** Lorena S. Pacheco, David A. Hernández-Ontiveros, Esmeralda Iniguez-Stevens, Stephanie Brodine, Richard S. Garfein, Margarita Santibañez, Miguel A. Fraga

**Affiliations:** 1 Department of Family Medicine and Public Health, School of Medicine, University of California, San Diego, La Jolla, CA USA; 2 Facultad de Medicina y Psicología, Universidad Autónoma de Baja California, Tijuana, Baja California Mexico; 3 School of Public Health, San Diego State University, San Diego, CA USA; 4 Division of Infectious Diseases and Global Public Health, School of Medicine, University of California San Diego, La Jolla, CA USA

**Keywords:** Type 2 diabetes mellitus, Metabolic syndrome, Prevalence, Rural population, Indigenous groups, Public health

## Abstract

**Background:**

Diabetes is a leading cause of morbidity and mortality in Mexico and understudied among indigenous populations. This study aimed to determine the prevalence and identify correlates of Type 2 diabetes mellitus (Type 2 DM) and metabolic syndrome (MetS) in a rural, indigenous community in Northwestern Mexico.

**Methods:**

A cross-sectional study was conducted in the community of San Quintin, Baja California, Mexico, among a sample of households. A total of 275 participants (≥18 years old) underwent a questionnaire, physical examination, and serologic test. Prevalence and adjusted odds ratio (AOR), using logistic regression modeling, were estimated with 95% confidence intervals (95% CI).

**Results:**

The prevalence of Type 2 DM and MetS was 21.8 and 53.1%, respectively. Mean ± standard deviation (SD) age and body mass index of study participants was 35.8 ± 13.0 years and 28.7 ± 5.6 kg/m^2^, respectively. Participants were 75% female and 60.7% self-identified as indigenous. Thirty-seven percent of adults had high blood pressure. After controlling for age, higher educational attainment had a protective effect on Type 2 DM (AOR = 0.39; 95% CI 0.20, 0.77). Additionally, the presence of MetS was associated with being female (AOR = 2.27; 95% CI 1.23, 4.14) and having lower educational attainment (AOR = 0.62; 95% CI 0.37, 0.94).

**Conclusions:**

The prevalence of Type 2 DM and MetS was high in this rural and indigenous population, and education was shown to play a critical role. These findings support the need for community-inclusive health-promoting interventions in rural communities.

## Background

Type 2 diabetes mellitus (Type 2 DM) and metabolic syndrome (MetS) are global public health problems [1, 2]. Type 2 DM is a chronic disease with hyperglycemia as a hallmark [2]. MetS refers to a cluster of factors including central obesity, hyperglycemia, hypertriglyceridemia, low plasma high-density lipoprotein (HDL)-cholesterol and hypertension, where the presence of at least three of these factors indicates MetS [3]. Entities including the National Cholesterol Education Program Adult Treatment Panel III (ATP III), the World Health Organisation and the International Diabetes Federation (IDF), have developed MetS diagnosis guidelines [4–6]. MetS components underlie cardiometabolic diseases increasing the risk of developing diabetes and cardiovascular disease (CVD), and even mortality [7, 8]. It has been shown that there is a five-fold increased risk in Type 2 DM development among those with MetS [9].

The prevalence of these two conditions continues to increase in parallel. Diabetes prevalence has almost doubled in the last 35 years globally, and it is estimated that between 10 and 40% of the world’s adult population has MetS [9, 10]. In Latin America, there is a MetS prevalence (weighted mean) of 24.9% (range: 18.8–43.3%) [11]. According to data from the 2006 Mexican National Health and Nutrition Survey (ENSANUT 2006) the prevalence of MetS could be as high as 49.8% in the overall adult population [12]. Mexico has the fifth highest number of individuals with diabetes, after China, India, the United States and Brazil [9], with diabetes accounting for the leading cause of morbidity [13]. The ENSANUT-Medio Camino 2016, a mid-point national survey, determined the diabetes prevalence to be 9.4% nationally, and 9.6% in rural northern areas of Mexico [14].

There has been limited research on Type 2 DM and cardiovascular risk factors in Mexican indigenous populations, and even less emphasis on MetS. A 2007 cross-sectional study found a prevalence of Type 2 DM in an indigenous population in the central valley of Mexico of 4.4% [15]. Another study found a prevalence of Type 2 DM among indigenous groups in neighboring Southern Mexican states of 8.7% among Zapotec and 6.9% among Mixe groups [16]. Data from a 2008 study in a migrant Mixtec population in rural Northwestern Mexico, found the prevalence of MetS was 41.1%, while that of Type 2 DM was 26.2% [17].

The aim of this study was to determine the prevalence and correlates of Type 2 DM and MetS in adults in a rural, indigenous community in Baja California, Mexico, while addressing sex differences for burden of disease. The current study tackled some of the limitations of previous work in the same community, where the prevalence of Type 2 DM and MetS was determined using a convenience sample and medical records of self-identified Mixtec adults [17].

## Participants and methods

### Study design and protocol

This cross-sectional community-wide study was conducted in a rural community in San Quintin, Baja California, Mexico (2013–2014), led by *Viaje Interinstitucional de Integración Docente, Asistencial y de Investigación* (VIIDAI), a binational inter-institutional program involving two universities in the United States (University of California San Diego and San Diego State University [SDSU]) and one in Mexico (Universidad Autónoma de Baja California ([UABC]). VIIDAI faculty and students operate a weekend-long clinic twice a year, where primary care services are offered to underserved community residents. San Quintin is an agriculture-centered municipality located about 300 km south of the United States-Mexico border on the west coast of the Baja California Peninsula. Since agriculture plays a key role in the town’s economy, it attracts farmers from South Mexico, primarily Oaxaca, in search of year-round work; the majority self-identify as indigenous. According to the latest surveillance data from Mexico’s Indigenous Infrastructure Program, there were 3805 persons living in the rural community we studied, of which 51% were adults [18].

Formative research, six months prior to the study start date, involved geographical information system (GIS) mapping, supplemented by fieldwork to confirm all edifices, to produce a map from which to sample residences for the current study. The sampling frame consisted of an enumerated list of 779 residences successfully identified as occupied households, of which 340 sampling units (i.e., homes) were randomly pre-selected via simple random sample selection. This approach was taken to maximize study efficiency, using study personnel wisely while capturing a representative sample of homes from all sections of the community.

The study protocol consisted of visiting each pre-selected household and requesting an adult to be screened for eligibility. Only one adult per household was allowed to participate in the study therefore a die (highest number rolled) was used to select the participant. Eligibility criteria included residing in the neighborhood for ≥6 months, age ≥ 18 years old, and agreement to participate in the study. Before the comprehensive examination, written informed consent in Spanish was obtained from all participants. The examination took place in mobile units stationed within two blocks of participants’ residence and involved an interviewer-administered questionnaire, anthropometric measurements, and blood sample. Following the examination, participants attended a nutritional counseling session and received $100.00 pesos (Peso Mexicano) as supplementary nutritional support. The research team was trained on the study protocol and followed a culturally-sensitive approach.

The study procedures were approved by the Bioethics Committee of the Facultad de Medicina y Psicología of UABC and by the Institutional Review Board of SDSU’s Human Research Protection Program.

### Socio-demographic and lifestyle questionnaire

Participants were interviewed in private by trained and native Spanish-speaking surveyors, following a standardised script and validated 45-item questionnaire to obtain socio-demographic and lifestyle data [19]. Socio-demographic questions included age, sex, occupation, years living in San Quintin, literacy, and number of school years completed. Lifestyle questions addressed physical activity, smoking habits, and alcohol consumption. Physical activity was assessed by a composite score of four variables, each graded from one (least activity) to four (most activity), for a maximum score of sixteen points. Variables ranked from least to most activity, included: (1) main mode of transportation: automobile, motorcycle, bicycle, walking; (2) physical activity intensity at work: sitting, standing and walking, carrying light load/climbing stairs, heavy work; (3) frequency of recreational activity (e.g. sport): none or rarely, 2–3 times/month, 1–2 times/week, and > 3 times/week); and (4) hours per week of watching television: > 13 h, 8–12 h, 4–7 h and < 3 h.

### Anthropometric and clinical measurements

Height, weight, waist circumference, and blood pressure, were measured by trained research assistants following Mexican public health and clinical guidelines [20, 21]. Height and weight were determined to the nearest 0.1 cm and 0.1 kg, respectively, using a mobile combined scale and stadiometer (Healthteam, GA, USA). Waist circumference was measured to the nearest 0.1 cm using a standard, non-stretch measuring tape (Gulik, Lafayette Instrument Co, IN, USA). Body mass index (BMI) was calculated as weight in kilograms divided by height in meters squared.

Blood pressure was obtained using a new digital blood pressure monitor (Omron 7 series Plus) from participants in a seated position after resting for 10 min, with arms relaxed and palms facing upward. Two readings were taken 5 min apart and the average of the two readings was recorded. If the difference between the first and the second reading was ≥10 mmHg for systolic blood pressure (SBP) and/or ≥ 6 mmHg for diastolic blood pressure (DBP), then a third measurement was done, and the average of all three measurements was calculated.

Anthropometry and blood pressure training was led by a study physician, certified dietitian-nutritionist and project researcher.

### Biochemical assays

A non-fasting venous blood sample of 5 cc was obtained from each participant. Assays included triglycerides, total cholesterol, and both low-density lipoprotein (LDL)- and HDL-cholesterol. The CardioChek PA (Polymer Technology Systems Inc., IN, USA) determined the lipid panel with 40 μl of whole blood.

Glycated hemoglobin (HbA1c) was measured using the rapid diagnostic and point of care NycoCard HbA1c Reader II (Alere, MA, USA**),** utilising 5 μl of blood from a finger stick sample. Both devices were calibrated every 10 tests.

### Outcomes measures

Type 2 DM was defined as HbA1c ≥6.5% according to American Diabetes Association (ADA) diagnosis criteria [22]. Participants that met the ADA’s diagnosis criteria for Type 2 DM were counseled by a physician and their test results were explained. A nutritional counseling session with a registered dietitian-nutritionist followed. Additionally, a sealed envelope that included a copy of their laboratory results and a clinical summary was provided to each participant, to take to their primary care physician for follow-up treatment and management.

MetS was defined using the IDF criteria [6]. This included participants with ethnic-specific waist circumference cut-offs of ≥90 cm in men and ≥ 80 cm in women. Additionally, participants had to meet at least two of the following to criteria: 1) elevated serum triglycerides (≥1.7 mmol/L); 2) low HDL-cholesterol levels (<1.03 mmol/L in men and <1.20 mmol/L in women); 3) elevated SBP ≥130 mmHg or DBP ≥85 mmHg; and 4) elevated fasting serum glucose levels, (≥5.6 mmol/L), or previously diagnosed diabetes.

### Statistical analysis

Sample characteristics were expressed as mean ± standard deviation (SD) for continuous variables, and frequencies and percentages for categorical variables. Prevalence of Type 2 DM and MetS, and cardiovascular risk factors were stratified by sex. Student’s t tests, and Pearson’s Chi-square tests were used to assess the associations between each factor and binary outcome variables. For analyses, completed school years was dichotomised (<6 years vs ≥6 years) based on mean of five schooling years, and income was dichotomised (<$3200 vs ≥ $3200) based on median earnings.

Separate multivariable logistic regression analyses were performed to identify factors independently associated with Type 2 DM and MetS. Backward stepwise elimination was used to remove non-significant variables until only statistically significant variables remained in the final model. Unadjusted and adjusted odds ratios and corresponding 95% confidence intervals were computed using simple and multivariable logistic regression analysis. Goodness of fit for the models was determined with the Hesmer-Lemeshow test. A *p* < 0.05 denoted statistical significance. SAS version 9.2 (SAS Institute) was used for all statistical analyses.

## Results

A total of 294 adults participated, corresponding to an 86% response rate. Ineligibility of participants and/or unresponsiveness explain the remaining 14%. Nineteen participants were excluded because of incomplete data, leaving 275 participants for statistical analysis.

Mean ± SD age was 35.8 ± 13.0 years, 75% of participants were women, close to two thirds of surveyed adults were born in the state of Oaxaca and, on average, had lived in San Quintin for almost two decades (Table 1). Number of completed school years was significantly lower among women (*p* = 0.004), and almost 82% of the study population was literate, with a significant sex difference (*p* = 0.016). Although the majority of women were homemakers, nearly 40% worked as field laborers. Sixty percent of study participants self-identified as having an indigenous ethnic background.

**Table 1:** Socio-demographic, lifestyle, clinical, and laboratory characteristics, stratified by sex, among adults in San Quintin, Baja California, Mexico – 2013-2014 (*n* = 275)

Characteristics^a^	Total (*n* = 275)	Men (*n* = 70)	Women (*n* = 205)	*p*-value*
Socio-demographic				
Age (years)	35.8 ± 13.0	37.0 ± 13.6	35.4 ± 12.8	0.367
Birth state, (%)				0.704
Born in Oaxaca	178 (64.7)	48 (68.6)	130 (63.4)	
Born in Baja California	47 (17.1)	10 (14.3)	37 (18.1)	
Other	50 (18.2)	12 (17.1)	38 (18.5)	
Income^b^ (pesos, monthly), (%)				0.887
0–2399	55 (20.0)	12 (17.1)	43 (21.0)	
2400-3199	69 (25.1)	17 (24.3)	52 (25.4)	
3200-4799	73 (26.5)	20 (28.6)	53 (25.9)	
4800-18,000	78 (28.4)	21 (30.0)	57 (27.8)	
Finished school^c^ (years)	4.8 ± 4.0	6.0 ± 4.0	4.4 ± 3.9	**0.004**
Ethnic group, (%)				0.673
Mestizo	108 (39.3)	26 (37.1)	82 (40.0)	
Indigenous	167 (60.7)	44 (62.9)	123 (60.0)	
Indigenous group^c^, (%)				0.583
Mixtec	95 (62.1)	24 (58.5)	71 (63.4)	
Other	58 (37.9)	17 (41.5)	41 (36.6)	
Literate, (%)				**0.016**
Yes	225 (81.8)	64 (91.4)	161 (78.5)	
No	50 (18.2)	6 (8.6)	44 (21.5)	
Speaks indigenous language^c^, (%)				0.312
Yes	107 (39.2)	31 (44.3)	76 (36.4)	
No	166 (60.8)	39 (55.7)	127 (62.6)	
Occupation, (%)				**< 0.0001**
Field laborer	130 (47.3)	49 (70.0)	81 (39.5)	
Home maker	107 (38.9)	3 (4.3)	104 (50.7)	
Other	38 (13.8)	18 (25.7)	20 (9.8)	
Residence in San Quintin (years)	18.1 ± 8.5	18.1 ± 9.4	18.1 ± 8.1	0.949
Lifestyle				
Current smoking status, (%)				**< 0.0001**
Yes	21 (7.6)	16 (22.9)	5 (2.4)	
No	254 (92.4)	54 (77.1)	200 (97.6)	
Alcohol intake, (%)				**< 0.0001**
Occasionally or more	31 (11.3)	21 (30.0)	10 (4.9)	
Rarely or never	244 (88.7)	49 (70.0)	195 (95.1)	
Total activity score	9.0 ± 2.6	9.1 ± 2.9	9.0 ± 2.5	0.667
Clinical				
Weight (kg)	65.0 ± 12.7	69.0 ± 13.4	63.6 ± 12.2	**< 0.0001**
Height (cm)	150.8 ± 8.9	159.5 ± 6.1	147.8 ± 7.6	**< 0.0001**
Body mass index (kg/m^2^)	28.7 ± 5.6	27.1 ± 4.9	29.2 ± 5.8	**0.006**
Waist circumference (cm)	96.1 ± 12.6	91.8 ± 10.4	97.6 ± 12.9	**< 0.0001**
Blood pressure, systolic (mmHg)	123.7 ± 18.8	131.2 ± 15.3	121.1 ± 19.3	**< 0.001**
Blood pressure, diastolic (mmHg)	78.8 ± 10.4	81.4 ± 7.7	77.9 ± 11.1	**0.005**
Laboratory				
Total cholesterol (mmol/L)	4.1 ± 0.9	4.0 ± 1.0	4.1 ± 0.9	0.437
HDL cholesterol (mmol/L)	1.1 ± 0.3	1.0 ± 0.3	1.1 ± 0.3	**0.002**
LDL cholesterol (mmol/L)	2.1 ± 0.7	2.2 ± 0.8	2.1 ± 0.6	0.650
Triglycerides (mmol/L)	2.0 ± 1.1	2.1 ± 1.1	1.9 ± 1.0	0.169
Glycated hemoglobin (A1c, %)	6.1 ± 1.4	6.1 ± 1.4	6.2 ± 1.5	0.524

Abbreviations: *kg* kilograms, *cm* centimeters, *mmHg* millimeters of mercury, *mmol/L* millimoles per liter

^a^Continuous variables are reported as mean ± standard deviation. Categorical variables are reported as number of participants and percentage in parenthesis

^b^Official currency of Mexico: Peso Mexicano

^c^Due to missing values, not all frequencies add up to 100%. Missing observations: Finished school *n* = 8; Indigenous group *n* = 14; Speaks indigenous language n = 2

^*^T-test or Chi Square Test for *p* values. Significant values (*p* < 0.05) are shown in bold text

Seven percent of the population reported consuming alcohol 2–3 times per month, with beer being the most frequently consumed alcoholic beverage. Only 8% of participants were current smokers, significantly higher in men (22.9%) compared to women (2.4%) (*p* < 0.001). The mean ± SD physical activity score was 9.0 ± 2.6 and did not differ by sex.

Mean ± SD BMI was 28.7 ± 5.6 kg/m^2^, however, women had a significantly higher BMI compared to men (*p* = 0.006). Obesity (BMI ≥30 kg/m^2^) was observed in 25.7% of men and 40.5% of women. There was no sex difference by total and LDL-cholesterol, and triglyceride levels, yet men had significantly lower HDL-cholesterol (*p* = 0.002). The mean ± SD HbA1c for the sample was 6.1 ± 1.4%, with no sex difference.

Prevalence of Type 2 DM and MetS was 21.8 and 53.1%, respectively (Table 2). Both conditions were higher in women; however, the difference by sex was only statistically significant for MetS (*p* = 0.011). Participants self-identified as having an indigenous background had a 19 and 53% prevalence of Type 2 DM and MetS, respectively; yet, there was no significant difference with those who identified as being mestizo. A large waist circumference was found in 80.7% of the population, significantly higher in women (*p* < 0.001), where more than 90% of adult women had a waist girth ≥80 cm. Eight percent of adults had hypertension and 28.7% had normal-high blood pressure, and more than half of adults displayed dyslipidemia.

**Table 2:** Type 2 Diabetes Mellitus, Metabolic Syndrome, and criteria diagnosis, stratified by sex, among adults in San Quintin, Baja California, Mexico – 2013-2014 (*n* = 275)

Diagnosis^a^	Total (*n* = 275)	Men (*n* = 70)	Women (*n* = 205)	*p*-value^*^
Type 2 DM^b^	60 (21.8)	13 (18.6)	47 (22.9)	0.446
Metabolic Syndrome^c^	146 (53.1)	28 (40.0)	118 (57.6)	**0.011**
Large waist circumference^d^	222 (80.7)	36 (51.4)	186 (90.7)	**< 0.001**
Normal-high blood pressure^e^	79 (28.7)	25 (35.7)	54 (26.3)	0.135
Hypertension^f^	22 (8.0)	6 (8.6)	16 (7.8)	0.838
Hypertriglyceridemia^g^	144 (52.4)	39 (55.7)	105 (51.2)	0.516
Low HDL cholesterol^h^	187 (68.0)	42 (60.0)	145 (70.7)	0.097

Abbreviations: *Type 2 DM* Type 2 Diabetes Mellitus, *HDL* high-density lipoprotein

^a^n (%)

^b^Type 2 Diabetes Mellitus was defined as a glycated hemoglobin A1c ≥ 6.5%, according to the American Diabetes Association criteria

^c^Metabolic Syndrome was defined following the International Diabetes Federation criteria: Ethnic-specific central obesity as waist circumference ≥ 90 cm in men and ≥ 80 cm in women for this population, plus any two of the following: elevated triglycerides ≥1.7 mmol/L; reduced HDL-cholesterol < 1.03 mmol/L in men and < 1.20 mmol/L in women; elevated blood pressure as systolic blood pressure ≥ 130 mmHg or diastolic blood pressure  85 ≥mmHg; and previously diagnosed with Type 2 diabetes mellitus

^d^Large waist circumference was defined as ≥90 cm in men and ≥ 80 cm in women

^e^Normal-high blood pressure was defined as systolic blood pressure 130–139 mmHg or diastolic blood pressure 85–89 mmHg, according to the Norma Oficial Mexicana criteria

^f^Hypertension was defined as having a blood pressure ≥ 140/90 mmHg, according to the Norma Oficial Mexicana criteria

^g^Hypertriglyceridemia was defined as triglycerides ≥1.7 mmol/L

^h^Low HDL cholesterol was defined as HDL cholesterol < 1.03 mmol/L in men and < 1.20 mmol/L in women

^*^Chi square test for *p* values. Significant values (*p* < 0.05) are shown in bold text

In bivariate analyses, age, sex, schooling, and literacy were associated with Type 2 DM (Table 3); while age, sex, schooling, literacy and birth state were associated with MetS (Table 4).

**Table 3:** Socio-demographic and lifestyle characteristics by Type 2 Diabetes Mellitus^a^ diagnosis among adults in San Quintin, Baja California, Mexico – 2013-2014 (*n* = 275)

Characteristic	Type 2 DM (*n* = 60)	No Type 2 DM (*n* = 215)	OR (95% CI)	*p*-value^*^
Socio-demographic				
Sex, (%)				
Male	13 (21.7)	57 (26.5)	1.00	
Female	47 (78.3)	158 (73.5)	1.30 (0.66, 2.59)	0.446
Age (years)	43.5 ± 14.6	33.6 ± 11.6	1.06 (1.03, 1.08)	**< 0.0001**
Birth state, (%)				0.130
Other	15 (25.0)	35 (16.3)	1.00	
Born in Oaxaca	39 (65.0)	139 (64.7)	0.66 (0.33, 1.32)	0.725
Born in Baja California	6 (10.0)	41 (19.0)	0.34 (0.12, 0.99)	0.068
Income^b^ (pesos, monthly), (%)				
< 3200	30 (50.0)	94 (43.7)	1.00	
≥ 3200	30 (50.0)	121 (56.3)	0.78 (0.44, 1.39)	0.387
Finished school^c^, (%)				
< 6 y (Less than Elementary)	46 (76.7)	102 (47.4)	1.00	
≥ 6 y (Elementary or More)	14 (23.3)	113 (52.6)	0.27 (0.14, 0.53)	**< 0.001**
Ethnic group, (%)				
Mestizo	28 (46.7)	80 (37.2)	1.00	
Indigenous	32 (53.3)	135 (62.8)	0.68 (0.38, 1.21)	0.185
Indigenous group^c^, (%)				
Other	11 (35.5)	47 (38.5)	1.00	
Mixteco	20 (64.5)	75 (61.5)	1.14 (0.50, 2.59)	0.755
Literate, (%)				
No	20 (33.3)	30 (14.0)	1.00	
Yes	40 (66.7)	185 (86.0)	0.32 (0.17, 0.63)	**0.001**
Speaks in indigenous language^c^, (%)				
No	37 (63.8)	129 (60.0)	1.00	
Yes	21 (36.2)	86 (40.0)	0.85 (0.47, 1.55)	0.600
Occupation, (%)				0.360
Other	8 (13.3)	30 (14.0)	1.00	
Field laborer	24 (40.0)	106 (49.3)	0.85 (0.35, 2.08)	0.340
Home maker	28 (46.7)	79 (36.7)	1.33 (0.55, 3.24)	0.248
Residence in San Quintin (years)	19.9 ± 8.3	17.6 ± 8.5	1.03 (0.99, 1.07)	0.080
Lifestyle				
Smoking status, (%)				
No	53 (88.3)	201 (93.5)	1.00	
Yes	7 (11.7)	14 (6.5)	1.90 (0.73, 4.93)	0.180
Alcohol intake^d^, (%)				
Rarely or never	53 (88.3)	191 (88.8)	1.00	
Occasionally or more	7 (11.7)	24 (11.2)	1.05 (0.43, 2.57)	0.913
Total activity score	9.2 ± 2.8	9.0 ± 2.6	1.03 (0.93, 1.15)	0.561

Abbreviations: *Type 2 DM* Type 2 Diabetes Mellitus, *OR* odds ratio, *CI* confidence interval, *y* years

^a^Type 2 Diabetes Mellitus was defined as a glycated hemoglobin A1c ≥6.5%, according to the American Diabetes Association criteria

^b^Official currency of Mexico: Peso Mexicano

^c^Due to missing values, not all frequencies add up to 100%. Missing observations: School years *n* = 8; Indigenous group *n* = 14; Speaks in indigenous language n = 2

^d^Alcohol intake defined as ‘rarely or never’ includes responses: never/rarely and 2–3 times per month; ‘occasionally or more’ includes responses: 1–2 times per week and 3 times or more per week

^*^Chi square test and logistic regression used for *p* values. Significant values (*p* < 0.05) are shown in bold text

**Table 4:** Socio-demographic and lifestyle characteristics by Metabolic Syndrome^a^ diagnosis among adults in San Quintin, Baja California, Mexico – 2013-2014 (*n* = 275)

Characteristic	MetS (*n* = 146)	No MetS (*n* = 129)	OR (95% CI)	*p*-value*
Socio-demographic				
Sex, (%)				
Male	28 (19.2)	42 (32.6)	1.00	
Female	118 (80.8)	87 (67.4)	2.03 (1.17, 3.54)	**0.011**
Age (years)	39.6 ± 13.3	31.4 ± 11.2	1.01 (1.04, 1.08)	**< 0.0001**
Birth state, (%)				**0.017**
Other	30 (20.5)	20 (15.5)	1.00	
Born in Oaxaca	100 (68.5)	78 (60.5)	0.86 (0.45, 1.62)	0.147
Born in Baja California	16 (11.0)	31 (24.0)	0.34 (0.15, 0.79)	**0.005**
Income^b^ (pesos, monthly), (%)				
< 3200	68 (46.6)	56 (43.4)	1.00	
≥ 3200	78 (53.4)	73 (56.6)	0.88 (0.55, 1.42)	0.599
Finished school^c^, (%)				
< 6 y (Less than Elementary)	94 (64.4)	53 (41.9)	1.00	
≥ 6 y (Elementary or More)	52 (35.6)	75 (58.1)	0.40 (0.24, 0.65)	**0.0002**
Ethnic group, (%)				
Mestizo	58 (39.7)	50 (38.8)	1.00	
Indigenous	88 (60.3)	79 (61.2)	0.96 (0.59, 1.56)	0.870
Indigenous group^c^, (%)				
Other	33 (38.4)	25 (37.3)	1.00	
Mixteco	53 (61.6)	42 (62.7)	0.96 (0.50, 1.85)	0.894
Literate, (%)				
No	35 (24.0)	15 (11.6)	1.00	
Yes	111 (76.0)	114 (88.4)	0.42 (0.22, 0.81)	**0.008**
Speaks in indigenous language^c^, (%)				
No	84 (58.3)	82 (63.6)	1.00	
Yes	60 (41.7)	47 (36.4)	1.25 (0.77, 2.03)	0.377
Occupation, (%)				0.998
Other	20 (13.7)	18 (14.0)	1.00	
Field laborer	69 (47.3)	61 (47.3)	1.02 (0.49, 2.10)	0.984
Home maker	57 (39.0)	50 (38.7)	1.03 (0.48, 2.23)	0.950
Residence in San Quintin (years)	18.1 ± 8.3	18.1 ± 8.6	1.00 (0.97, 1.03)	0.984
Lifestyle				
Smoking status, (%)				
No	135 (92.5)	119 (92.2)	1.00	
Yes	11 (7.5)	10 (7.8)	0.97 (0.40, 2.37)	0.946
Alcohol intake^d^, (%)				
Rarely or never	131 (89.7)	113 (87.6)	1.00	
Occasionally or more	15 (10.3)	16 (12.4)	0.81 (0.38, 1.71)	0.577
Total activity score	8.8 ± 2.6	9.3 ± 2.6	0.91 (0.85, 1.03)	0.066

Abbreviations: *MetS* Metabolic Syndrome, *OR* odds ratio, *CI* confidence interval, *y* years

^a^Metabolic Syndrome was defined following the International Diabetes Federation criteria: Ethnic-specific central obesity as waist circumference ≥ 90 cm in men and ≥ 80 cm in women for this population, plus any two of the following: elevated triglycerides ≥1.7 mmol/L; reduced HDL-cholesterol < 1.03 mmol/L in men and < 1.20 mmol/L in women; elevated blood pressure as systolic blood pressure ≥ 130 mmHg or diastolic blood pressure ≥ 85 mmHg; and previously diagnosed Type 2 Diabetes Mellitus

^b^Official currency of Mexico: Peso Mexicano

^c^Due to missing values, not all frequencies add up to 100%. Missing observations: School years n = 8; Indigenous group n = 14; Speaks in indigenous language n = 2

^d^Alcohol intake defined as ‘rarely or never’ includes responses: never/rarely and 2–3 times per month; ‘occasionally or more’ includes responses: 1–2 times per week and 3 times or more per week

^*^Chi square test used for *p* values. Significant values (*p* < 0.05) are shown in bold text

After controlling for age, those who completed at least elementary school were almost 60% less likely to have Type 2 DM (adjusted OR [AOR] = 0.39, 95% CI 0.20, 0.77) (Table 5). Women had 2.27 (95% CI: 1.23, 4.14) higher odds of MetS compared to men, after adjusting for age and education. Furthermore, after controlling for sex and age, completion of at least elementary school was inversely associated with MetS (AOR = 0.62, 95% CI 0.37, 0.94).

**Table 5:** Multivariable logistic regression^a^ analyses of Type 2 Diabetes Mellitus^b^ and Metabolic Syndrome^c^ in adults in San Quintin, Baja California, Mexico – 2013-2014 (*n* = 275)

	Type 2 Diabetes Mellitus^b^				Metabolic Syndrome^c^			
	Full Model		Final Model		Full Model		Final Model	
Characteristic	OR (95% CI)	*p*-value^*^	OR (95% CI)	*p*-value^*^	OR (95% CI)	*p*-value^*^	OR (95% CI)	*p*-value^*^
Age	1.05 (1.02, 1.08)	**0.0002**	1.05 (1.03, 1.07)	**< 0.0001**	1.06 (1.03, 1.09)	**< 0.0001**	1.06 (1.03, 1.08)	**< 0.0001**
Women^d^	1.25 (0.59, 2.66)	0.567	–	–	2.27 (1.23, 4.20)	**0.009**	2.27 (1.23, 4.14)	**0.009**
≥6 y of schooling (Elementary)^e^	0.42 (0.20, 0.88)	**0.005**	0.39 (0.20, 0.77)	**0.007**	0.59 (0.33, 1.00)	**0.051**	0.62 (0.37, 0.94)	**0.050**
Literate^f^	0.84 (0.38, 1.85)	0.665	–	–	1.11 (0.50, 2.42)	0.798	–	–
Born in Oaxaca^g^	N/A				0.62 (0.31, 1.25)	0.385	–	–
Born in BC^g^	N/A				0.65 (0.26, 1.62)	0.644	–	–
			GOF	0.801			GOF	0.323

Abbreviations: *y* years, *BC* Baja California, *OR* odds ratio, *CI* confidence interval, *GOF* Goodness of Fit Test, -- not included in the final model

^a^Logistic regression modeling, presenting odds ratio and 95% confidence interval

^b^Type 2 Diabetes Mellitus was defined as a glycated hemoglobin A1c ≥6.5%, according to the American Diabetes Association criteria

^c^Metabolic Syndrome was defined following the International Diabetes Federation criteria: Ethnic-specific central obesity as waist circumference ≥ 90 cm in men and ≥ 80 cm in women for this population, plus any two of the following: elevated triglycerides ≥1.7 mmol/L; reduced HDL-cholesterol < 1.03 mmol/L in men and < 1.20 mmol/L in women; elevated blood pressure as systolic blood pressure ≥ 130 mmHg or diastolic blood pressure ≥ 85 mmHg; and previously diagnosed Type 2 Diabetes Mellitus

^d^Reference: Men

^e^Reference: <6 y of schooling (No Elementary)

^f^Reference: Illiterate

^g^Reference: Other

^*^P values calculated by logistic regression modeling, adjusted for all other listed variables in each of the models (full and final). Significant values (*p* < 0.05) are shown in bold text

## Discussion

This study found a Type 2 DM and MetS prevalence in adults in a rural indigenous community in San Quintin, Mexico, of 21.8 and 53.1%, respectively, with a higher prevalence of both conditions in women.

Prevalence of Type 2 DM in the community was high. The latest national survey results found a diabetes prevalence of 9.6% in northern rural areas of Mexico [23], much lower than our results. Escobedo et al. and Alvarado-Osuna et al., determined diabetes prevalence in indigenous groups in Southern Mexico, with our study population showing a much higher prevalence than that observed in Zapotec (8.7%), Mixe (6.9%) [16] and Otomi (4.4%) [15] groups. Zapotec and Mixe women had a higher diabetes prevalence, 13.3 and 7.1%, respectively, when compared to Zapotec and Mixe men, with a 6.2 and 5.7% prevalence, respectively. Our study shared the commonality of a higher proportion of women (22.9%) with Type 2 DM, in contrast to men (18.6%), yet recognize the larger prevalence of the condition in our sample of rural indigenous adults. Compared to the findings reported by Goodman et al., in the same community eight years prior (26.2%), the prevalence of Type 2 DM was reduced by 16.8% [17]. Goodman et al., defined Type 2 DM as having a HbA1c of ≥6.5% or a fasting glucose of ≥126 mg/dL; it was unclear of the proportion of those defined by either HbA1c or fasting glucose.

Similarly, we found a very high MetS prevalence. MetS was 29% higher than what was previously found in the same community in 2008 (41.1%) [17]. We are cautious when making direct comparisons because the previous study followed the ATP III guideline criteria, while we used the IDF definition, in which central obesity, using ethnic-specific waist circumference cut-offs, is a requirement. Our findings were similar to the ENSANUT 2006 MetS prevalence of 49.8% [12], and comparable to a recent Northeastern Mexico study that reported an overall MetS prevalence of 54.8% in the state of Nuevo Leon [24], with closer resemblance to the IDF definition. Although this Northeastern Mexico study included an older population (mean ± SD age 51.0 ± 19.0 and 50.7 ± 19.0 years, men and women, respectively), teenagers were also included. Considering the MetS prevalence in Latin America, our findings sit outside the estimated range of 18.8–43.4% [11].

A higher prevalence of MetS was observed in women when compared to men (56.7% vs 40.0%), analogous to the Northeastern Mexico study findings (60.4% in women vs 48.9% in men) [24]. This was further observed in our multivariable model results, when after adjusting for covariates, women had a 2.27 (95% CI 1.23, 4.14) greater odds of MetS compared to men. The 40% MetS prevalence in men was akin to the general prevalence reported by Ramirez-Vargas et al., in a sample of Oaxacan men living in rural and urban communities (40.0% in rural vs 41.2% in urban) [19]; yet our results were 31% higher than the prevalence of MetS among those Oaxacan men living in a rural area (27.6%). Regardless of the differences between studies, our results indicate that the prevalence of MetS was high in our study population and has increased since the last cross-sectional examination.

Schooling was independently associated with both Type 2 DM (AOR = 0.39, 95% CI 0.20, 0.77) and MetS (AOR = 0.62, 95% CI 0.37, 0.94). Recent Type 2 DM epidemiological surveillance data highlighted that among all identified cases for the 2016 year, almost 50% had less than elementary school education, with less cases having had at least elementary school [25]. The ENSANUT 2006 national results reported that the lower the education level, the higher prevalence of MetS [26]. Those adults with less than elementary school education had the highest prevalence of MetS, 60.1% (95% CI 56.9–63.3%) while those with more than high school attainment had 39.3% (95% CI 32.5–46.6%) prevalence of MetS in 2006. This evidence supports our findings, underscoring educational level as an important socio-demographic characteristic and possible protective factor for Type 2 DM and MetS in indigenous Mexican groups.

The population also exhibited altered cardiovascular characteristics. A large waist circumference was observed in a large proportion of the study population, indicating excess abdominal body fat and health consequences associated with central obesity. Intra-abdominal fat, MetS and Type 2 DM are inter-related [27], thus having an elevated intra-abdominal fat accumulation may have a direct function in the development of MetS [28] and Type 2 DM, because of its association with insulin resistance [27].

More than a third of participants were hypertensive or had normal-high blood pressure, worrisome since elevated blood pressure is a silent killer and major risk factor for CVD. The new hypertension guidelines acknowledge complications at lower levels of blood pressure, thus aggressive screening should be highlighted [29]. More than half of adults presented dyslipidemia, similar to adults in Northeastern Mexico, were two thirds had low HDL-cholesterol levels and almost half had elevated triglycerides [24].

The mean ± SD total activity score for the population was 9.0 ± 2.6, with no sex difference. This translates to an average level of total activity based on transportation, television watching time, occupation-related intensity and recreational physical activity. We understand the relationship between physical activity and Type 2 DM and MetS, yet we did not see this in the present study.

We acknowledge the cross-sectional nature of the study design and related shortcomings, yet our study aim consisted of determining Type 2 DM and MetS prevalence while describing potential disease correlates in this particular population, without a singular exposure-disease relationship. Thereby, study limitations include self-reported lifestyle measures and incomplete information on family history, introducing information bias. Dietary intake was not assessed, and likelihood of residual confounding is recognised. Generalisability is restricted to neighboring communities with a demographically similar population.

Study strengths include the use of GIS mapping and systematic random sampling of households, which allowed us to obtain a more representative sample of the community than prior studies. The application of a detailed protocol performed by a rigorously trained team in a rural and inaccessible area is a notable attainment, while determining health outcomes with standardised and validated objective measures, applied in a global public health setting. Diabetes in on the rise in Mexico, thereby we consider our findings to be relevant and timely. We are contributing to the study of Mexican rural and indigenous communities, whilst providing crucial evidence to help inform interventions and health policy.

## Conclusions

Findings elucidate the elements of Type 2 DM and MetS in this rural population, while enhancing the literature on these two conditions and cardiometabolic risk factors in indigenous communities in Mexico. It is important to highlight that our study aimed to describe the magnitude of the burden of Type 2 DM and MetS, the latter serving as an indicator of the number of additional community members at risk of Type 2 DM. Comprehensive community-inclusionary interventions should be pursued to help prevent and mitigate potential health consequences related to these conditions, especially including women. Education must be considered as a key factor when addressing the prevention of Type 2 DM and MetS.

## Abbreviations

95% CI: 95% confidence interval
AOR: Adjusted odds ratio
ATP III: Adult treatment panel III
BMI: Body mass index
CVD: Cardiovascular disease
DBP: Diastolic blood pressure
ENSANUT: Encuesta Nacional de Salud y Nutrición (Spanish), Mexican National Health & Nutrition Survey (English)
HbA1c: Glycated hemoglobin
HDL-cholesterol: High density lipoprotein cholesterol
IDF: International diabetes federation
LDL-cholesterol: Low density lipoprotein cholesterol
MetS: Metabolic syndrome
SBP: Systolic blood pressure
SD: Standard deviation
SDSU: San Diego State University
Type 2 DM: Type 2 diabetes mellitus
UABC: Universidad Autonoma de Baja California
VIIDAI: Viaje Interinstitucional de Integración Docente, Asistencial y de Investigación (Spanish), Inter-institutional Field Experience for Integration, Teaching, Health Services and Research (English)
